# Mobile Health Initiatives in Vietnam: Scoping Study

**DOI:** 10.2196/mhealth.8639

**Published:** 2018-04-24

**Authors:** Jeffrey A Lam, Linh Thuy Dang, Ngoc Tran Phan, Hue Thi Trinh, Nguyen Cong Vu, Cuong Kieu Nguyen

**Affiliations:** ^1^ Brown University Providence, RI United States; ^2^ Institute of Population, Health and Development Hanoi Viet Nam

**Keywords:** mHealth, eHealth, mobile health, telemedicine, Vietnam, scoping review

## Abstract

**Background:**

Mobile health (mHealth) offers a promising solution to the multitude of challenges the Vietnamese health system faces, but there is a scarcity of published information on mHealth in Vietnam.

**Objective:**

The objectives of this scoping study were (1) to summarize the extent, range, and nature of mHealth initiatives in Vietnam and (2) to examine the opportunities and threats of mHealth utilization in the Vietnamese context.

**Methods:**

This scoping study systematically identified and extracted relevant information from 20 past and current mHealth initiatives in Vietnam. The study includes multimodal information sources, including published literature, gray literature (ie, government reports and unpublished literature), conference presentations, Web-based documents, and key informant interviews.

**Results:**

We extracted information from 27 records from the electronic search and conducted 14 key informant interviews, allowing us to identify 20 mHealth initiatives in Vietnam. Most of the initiatives were primarily funded by external donors (n=15), while other initiatives were government funded (n=1) or self-funded (n=4). A majority of the initiatives targeted vulnerable and hard-to-reach populations (n=11), aimed to prevent the occurrence of disease (n=12), and used text messaging (short message service, SMS) as part of their intervention (n=14). The study revealed that Vietnamese mHealth implementation has been challenged by factors including features unique to the Vietnamese language (n=4) and sociocultural factors (n=3).

**Conclusions:**

The largest threats to the popularity of mHealth initiatives are the absence of government policy, lack of government interest, heavy dependence on foreign funding, and lack of technological infrastructure. Finally, while current mHealth initiatives have already demonstrated promising opportunities for alternative models of funding, such as social entrepreneurship or private business models, sustainable mHealth initiatives outside of those funded by external donors have not yet been undertaken.

## Introduction

### Background

Vietnam has attained remarkable economic and health achievements in recent decades [1], but the health system still faces significant challenges. Vietnam has a high prevalence of communicable diseases coupled with an increase in the prevalence of noncommunicable diseases [2] and a high out-of-pocket payment rate (47% as of 2015 [3]), making up over one-third of all health care costs in Vietnam [4]. There are severe shortages in health resources, especially in remote regions, with only 7.9 physicians per 10,000 individuals overall in 2014 [5] and as few as 1 per 10,000 physicians in hard-to-reach areas [6]. These systemic challenges have encouraged the use of technology-based innovations in health care, particularly mHealth or “the use of mobile and wireless technologies to support the achievement of health objectives” [7].

mHealth initiatives remain unproven and many remain in early or pilot phases [8], despite the growing attention from researchers and policy makers as a means to confront health challenges in low-resource settings. A 2013 review of mHealth interventions in low- and middle-income countries found that few mHealth initiatives were operating at scale, and there was little evidence of formal evaluations of these initiatives [9]. Another mHealth review corroborated these findings and found a lack of significant investment in mHealth policy in low-resource environments due to a lacking evidence base. In the review, only, 15 out of 53 studies attempted to provide evidence of health care metrics such as improvements in health care processes or public health indicators [10]. The most common use of mHealth in low- and middle-income countries was phone reminders and one-way text message for follow-up appointments, data gathering, and encouragement of healthy behavior [9].

In Vietnam, the 2016 rate of mobile-cellular subscriptions as a percent of the population is 131.8, equating to more than 1 cell phone per person [11]. As of May 2017, Vietnam had about 58.9 million Internet users, which is more than half of the population [12]. SMS text messaging (SMS, short message service) is inexpensive and mobile phone service coverage is wide (eg, [13,14]).

### Objectives

While there are available research papers reviewing the state of mHealth in other low and middle-income countries [15,16], at the time of writing of this review, there were no studies summarizing the extent of mHealth research and interventions in Vietnam. mHealth is an emerging field in Vietnam, and many of the projects are still in pilot phases or are ongoing. As more initiatives start in Vietnam, it is necessary to standardize, share information, and develop a set of best practices for the country. In addition, as Vietnam is in the process of revising and rewriting its young eHealth policies, it is important to understand barriers and enablers to mHealth implementation. Aiming to advance policy and practices for mHealth in Vietnam, the goal of this scoping study is twofold: (1) to summarize the extent, range, nature, and location of mHealth initiatives in Vietnam and (2) to examine the opportunities and threats of mHealth utilization in the Vietnamese context.

## Methods

In this scoping study, we used Arksey and O’Malley’s (2005) [17] 5-step framework for scoping studies to retain methodological rigor and to allow the use of multimodal information sources, including published literature, gray literature (ie, government reports and unpublished literature from internal reports), conference presentations, Web-based documents, and key informant interviews. After identifying the study objectives (listed above), we identified relevant mHealth initiatives through electronic search and discussions with key stakeholders.

### Data Collection

#### Electronic Search

Electronic searches were performed to discover initiatives with internationally and publicly available information and publications. We conducted our literature search using the following databases and websites: PubMed MEDLINE, EMBASE, Cochrane Central Register of Controlled Trials (CINAHL) PsycINFO; Medline, Academic Search Premier, Global Health, Web of Science, ClinicalTrials.gov, World Health Organization clinical trials, and Google Scholar. The first strategy searched for the combined free-text words “mHealth” AND “Vietnam OR Viet Nam” in the title, abstract, and keywords. The second strategy searched for the combined free-text words “mobile phone$ OR cellphone$” AND “health” AND “Vietnam OR Viet Nam” in the title, abstract, and keywords. Search terms were limited to English words. The searches were initially completed by 2 authors (JL and LD) in January 2017 and were repeated in January 2018 upon revision of the manuscript. Duplicate records were removed, and 2 authors independently screened remaining records’ abstracts before proceeding to the full texts to decide for inclusion, classification, and further investigation. The criteria for a record study inclusion were if the record described an initiative that (1) utilized mobile technology in a health-related capacity and (2) was based in Vietnam.

#### Key Informant Interviews

To minimize publication bias, key informant interviews were performed after the electronic search to collect data from unpublished work or initiatives with information not publicly available [18]. After the electronic search was completed and relevant mHealth initiatives identified, we requested to speak to representatives from each of the initiatives, aiming to obtain initiative-specific information that was not publicly available. Additional interviews were also conducted with other key stakeholders in relevant government organizations such as the Vietnam Ministry of Health, nongovernmental organizations, private organizations, and key actors and researchers at leading health science institutions such as the Hanoi University of Public Health and Hanoi Medical University. The goal of these additional interviews was to discover and collect information from other unpublished initiatives. All interviews were conducted using a semistructured format in either English or Vietnamese, with the questions left intentionally open-ended as to not limit the emergence of any mHealth themes. All interviewees were asked if they knew of other mHealth initiatives in Vietnam, lessening the chance of undiscovered initiatives. All interviewees verbally consented to being interviewed, and all interviews were recorded.

### Initiative Data Analysis and Extraction

Data were charted concurrently as the electronic search and the initiative-specific interviews. As we received information from the initiatives, we used an integrative process to create a list of variables, which were continuously updated based on incoming information. The definitions of the extracted variables were agreed on by 2 authors (JL, CN). In all, 3 authors (JL, NP, and HT) independently extracted all relevant information from all available sources, including electronic search articles and initiative leader interviews recordings.

The final data collection form, consisting of data from both the electronic search and key informant interviews, included project description, type of data sources, intervention period, stage of project, continuity status of project, mHealth domain, disease area, targeted population group, type of the primary funding source, and project implementation location, as summarized in the Multimedia Appendix 1. Stage of the initiative was classified as either ongoing or completed, defined by the project being finished within their intended primary data collection period. Initiatives classified as completed initiatives were then categorized into continuing or noncontinuing initiatives, defined as still actively using the technology and infrastructure built during the initiative to recruit more participants. The mHealth domain was classified based on the 2015 International Telecommunication Union M-Powering Development Initiative document [19]. The broad groups included solutions across the patient pathway (prevention, diagnosis, treatment, monitoring) and health care systems strengthening (emergency response, health care practitioner support, health care surveillance, and health care administration). The provisioning technologies used were classified into the following groups: SMS, voice-based technology, app, Web-app, video-telephony, and other functionalities. Disease areas (ie, heart disease, HIV/AIDS) and population groups (ie, health care providers, migrant women) were broadly categorized into the specific disease and population group the initiative targeted. Finally, primary funding source was classified by initiatives’ main source of funding.

### Thematic Data Analysis for Key Informant Interviews

The thematic data analysis in this study involved a multistage process to develop and refine a codebook using information from the semistructured interviews with all key informants, including the initiative-specific leaders and other stakeholders. We used Young et al’s (2014) study on the development and refinement of a codebook to guide the analysis [20]. The first stage focused on familiarization with the data by listening and independently taking notes from the recordings. Thereafter, 2 authors (JL and CN) created an initial list of possible codes and definitions for these codes.

In the second stage, 3 authors (JL, NP, and HT) independently extracted comments from the semistructured interviews and classified all meaningful segments into these codes. We aimed to limit interpretation at this stage. Extractors were encouraged to refine the codebook, and inconsistencies in extraction were discussed with all extractors at the completion of this stage. We did not consider data saturation, as we aimed to capture as much data as possible by interviewing all relevant stakeholders we were able to find in Vietnam.

In all, 2 authors (JL and CN) collated the information and used thematic analysis techniques [21] to iteratively create the final codebook, which included themes relating to Vietnamese sociocultural factors, technological factors, and collaboration factors. Finally, initiative leaders who were interviewed were sent a draft of the paper in May 2017, with the opportunity to review and suggest edits.

## Results

### Data Sources Overview

The initial electronic database search retrieved 128 records. After duplication removal and screening, 27 records met inclusion criteria, which were sorted into 10 initiatives (Initiative #1, 4, 6, 7, 10, 11, 13, 16, 17, and 20). Of these 27 records, there were 12 research papers [13,14,22-31], 8 conference presentations [32-39], 2 Vietnamese governmental documents [40,41], 2 graduate dissertations [42,43], 2 case studies [44,45], and 1 project technical report [46].

A total of 14 interviews were conducted, in which 12 interviews were related to one or more of the initiatives and contributed to 10 more initiatives (Initiative #2, 3, 5, 8, 9, 12, 14, 15, 18, and 19). The other 2 interviews were conducted with relevant stakeholders providing an overview of mHealth in Vietnam. A total of 6 conference abstracts, which were not available publicly, were additionally provided by the mHealth initiative leaders [47-52].

### Stage, Continuity, and Funding

Five (Initiative #3, 5, 10, 12, and 19) out of the 20 initiatives were ongoing at the time of data collection, although one of these ongoing initiatives had already analyzed preliminary results (Initiative n#10). A total of 15 initiatives (Initiative #1, 2, 4, 6, 7, 8, 9, 11, 13, 14, 15, 16, 17, 18, and 20) were already completed.

Out of these 15 initiatives completed with their intended primary research and data collection period, 4 (Initiative #1, 9, 11, and 13) were continuing to use the materials, infrastructure, and technology created during their initiatives to engage with their end users at the time of our data collection. Two initiatives (Initiative #2 and 16) were not considered for continuity, as the goal was to examine feasibility and not to create sustainable initiatives. No information could be collected about the sustainability of 2 initiatives (Initiative #17 and 20), and the remaining 7 initiatives (Initiative #4, 6, 7, 8, 14, 15, and 18) were considered not continuing.

Two initiatives directly cited budgeting constraints and difficulties seeking out funding as a primary challenge (Initiative #9 and 18). A total of 4 initiatives stated failure to find funding as one of the primary causes for the initiative’s discontinuation (Initiative #6, 14, 16, and 17).

A vast majority of initiatives (n=15) were funded by external donors (Initiative #2, 3, 4, 5, 7, 8, 9, 11, 12, 13, 16, 17, 18, 19, and 20). In all, 4 initiatives were self-funded (Initiative #6, 10, 14, 15), and one was government funded (Initiative #1).

### mHealth Services in Vietnam

On the basis of the 2015 International Telecommunication Union (ITU) categorizations of mHealth, a majority of the initiatives (n=12) had some component of prevention or raising awareness and encouraging participants to adopt healthier behaviors (Initiative #3, 4, 5, 6, 7, 8, 9, 11, 13, 15, 17, and 18). For example, one of the first initiatives carried out in Vietnam in 2010 piloted the use of SMS in order to increase general health education for ethnic minority populations. This initiative reported an increase in self-reported positive health behaviors for the participants after the initiative, including adoption of safer sexual practices and quitting smoking (Initiative #7). Another initiative, carried out by the Hanoi University of Public Health, provided sexual and reproductive health services for female migrant workers via SMS and a free counseling hotline, which were demonstrated to increase women’s health knowledge (Initiative #17). Similar to these 2 initiatives, other initiatives classified in the prevention domain attempted to prevent disease by using various mHealth technologies to facilitate the dissemination of health information.

Health practitioner support or intelligent decision support systems for diagnosis, treatment, information lookup, or information dissemination, was a component of 8 initiatives (Initiative #1, 2, 3, 5, 6, 11, 13, and 16). For example, 2 initiatives sent SMS with the aim of improving the health knowledge of health practitioners (Initiative #2 and 16); however, the one completed initiative did not show increases in health knowledge post intervention (Initiative #16). The Government of Vietnam (GVN) started an initiative to build capacity for provincial satellite hospitals across the country utilizing telemedicine technology (Initiative #1). Other initiatives facilitated case management for health practitioners by allowing them easier access to information such as immunization records via a Web application in real time (Initiative #11) and case management support via SMS notifications (Initiative #13).

To address challenges in maintaining continual and consistent care to patients, 4 initiatives assisted with treatment aiming to improve medical adherence (Initiative #3, 14, 19, and 20). These 4 initiatives targeted tuberculosis (TB) medication adherence (Initiative #3), general medical adherence (Initiative #14), and antiretroviral therapy adherence (Initiative #19 and 20).

In all, 2 initiatives assisted with monitoring, which includes identifying illnesses and tracking vital changes in health parameters (Initiative #9 and 10). One initiative piloted the use of a mobile electrocardiogram (ECG) to allow heart disease patients a method to monitor their heart’s activity in real time outside of the hospital setting (Initiative #10). Another initiative conducted by the Center for Creative Initiatives in Health and Population developed a Web application for individuals with autism spectrum disorder (ASD), which has allowed parents to screen for ASD at an earlier phase and has used questionnaires for the parents to monitor the child’s progress (Initiative #9).

Finally, 2 initiatives displayed components of health surveillance, or the timely collection or transmission of health-data to bridge gaps between the commune, district, provincial, and national levels (Initiative #4 and 11). For example, a disease surveillance mHealth initiative aimed to upgrade an antiquated paper-based tracking system to help provide the GVN timely infectious disease statistics (Initiative #4). An immunization registration system also allowed health managers at higher health care levels to generate reports and plan immunization campaigns, reporting to save the Vietnamese government money by improving the old paper-based system (Initiative #11).

### Disease Areas and Populations Targeted

A majority of the initiatives (n=11) targeted disease areas associated with vulnerable and hard-to-reach populations. Maternal and reproductive health was the primary target of 4 of the initiatives. The end users for 2 of these initiatives were female migrants (Initiative #6 and 17), and the other 2 were ethnic minority women (Initiative #12 and 13). While 2 more initiatives addressed women and their children, 1 focused on infectious disease (Initiative #11), and the other targeted early ASD screening and tracking (Initiative #9). A total of 4 initiatives aimed to support treatment and care of persons living with HIV, with one of the initiatives focusing on adolescents living with HIV (Initiative #19) and one targeting people living with HIV (Initiative #20), and the remaining 2 initiatives targeting key affected populations (Initiative #8 and 18). Finally, one initiative targeted a nonspecific disease area, but the target population was ethnic minorities (Initiative #7).

There were 6 initiatives that did not specifically target a particular disease area. As mentioned above, one initiative targeted ethnic minorities (Initiative #7), 2 targeted nonspecific populations (Initiative #14 and 15), while the remaining 3 initiatives targeted health care providers (Initiative #1, 2, and 16), which aimed to build the overall capacity of the health care system. Additionally, there were 2 initiatives that addressed heart disease, both aiming to target those with high heart disease risk (Initiative #5 and 10). The last 2 initiatives were related to TB, with the end users being TB patients (Initiative #3) and infectious disease, with the end users being health care practitioners (Initiative #4). Overall, the end users of the mHealth initiative for 4 initiatives were health care practitioners, while the primary end users for the other 16 initiatives were aiming to create solutions across the patient pathway.

### Provisioning Technology

A few mHealth initiatives integrated multiple components of technology into their initiative, with 5 initiatives using more than one type of provisioning technology (Initiative #7, 8, 14, 17, and 19). The most popular provisioning technology in Vietnam was SMS, with 14 initiatives using SMS in some form (Initiative #2, 3, 4, 5, 6, 7, 8, 11, 13, 14, 16, 17, 18, and 19). In all, 6 initiatives used a Web application (Initiative #2, 3, 5, 9, 11, and 18), and 5 initiatives integrated voice calling into their projects (Initiative #7, 8, 14, 17, and 19). Video telephony (Initiative #1), mobile projectors (Initiative #15), mobile ECG device (Initiative #10), flashing light and sound reminders from pill bottle (Initiative #19), mobile phone app (Initiative #20), and tablet app (Initiative #12) were each used in one of the initiatives.

### Vietnamese and Sociocultural Factors

Key informants from 6 initiatives cited high mobile phone penetration (Initiative #4, 5, 13, 14, 17, and 18) as a strength of mHealth utilization in the Vietnamese context, while 7 key informants indicated strong potential to reach vulnerable participants as a strength (Initiative #2, 3, 6, 9, 14, 16, and 17). For example, the Hanoi University of Public Health conducted an mHealth initiative capable of reaching vulnerable migrant workers outside of normal working hours (Initiative #17).

Multiple sociocultural factors challenged the initiatives. Key informants from 2 initiatives indicated that Vietnamese family structure and cell phone sharing practices prevented SMS from reaching the targeted individuals (Initiatives #13 and 16). One key informant noted participants are more willing to use mobile technology than face-to-face meetings for taboo topics (Initiative #17). However, another key informant noted the use of mobile technology made participants more worried of the disclosure of confidential information (Initiative #14).

Another challenge to mHealth in Vietnam pertains to the numerous languages and dialects. Interviewees stated many of the individuals with the worst health outcomes do not speak Vietnamese, causing challenges for the initiatives targeting these non-Vietnamese speaking populations. Two initiatives translated their initiative material to ethnic minority languages (Initiative #7 and 12). Key informants from 2 initiatives stated diacritic marks used in the Vietnamese language presented a challenge for initiatives utilizing SMS, as many phones cannot correctly format these diacritic marks (Initiative #13 and 16). Consequently, initiatives commonly neglected the diacritic marks, which one initiative stated caused challenges for some participants who had trouble reading the SMS (Initiative #13).

### Technological Factors

Interviewees noted the strengths of mHealth technology across multiple domains within the health care system. From a research perspective, 3 interviewees stated technological systems facilitated researchers in managing systems by capturing more and higher quality data (Initiative #6, 9, and 11). Interviewees stated they thought mHealth led to improved management of patients and customizability of treatment (Initiative #13 and 19). For example, a project sending informational SMSs to mothers provided customized information to their participants based on the mothers’ current stage of motherhood (Initiative #13).

From a provider perspective, 3 initiatives noted that mHealth facilitated the tracking and management of patients, thereby saving man-hours in a human resource–deficient setting (Initiative #3, 15, and 18). However, one initiative pointed out mHealth may subsequently shift the burden of work from one human health resource to another. For example, an initiative aiming to distribute medical education texts to physician’s assistants noted the educational texts had to be generated by 6 health students in the United States and later reviewed by the principal investigator, requiring a significant amount of time from highly educated staff (Initiative #16).

Lack of technological infrastructure and technological glitches challenged mHealth initiatives. One initiative encountered technical difficulties with their toll-free number as a result of lack of connectivity between different service providers (Initiative #11). Another initiative reported participants were not familiar using toll-free numbers, as users were worried they would be charged for the calls (Initiative #7). One mHealth initiative struggled to retain and follow participants through the mHealth intervention period due to the tendency of Vietnamese users to use multiple numbers to call and text as a result of the cheap promotional SIM cards available in Vietnam (Initiative #17). Technological glitches were mentioned as a challenge for 3 initiatives (Initiative #2, 9, and 16).

### Collaboration Factors

Initiatives had difficulty finding the appropriate technological collaborators. One initiative mentioned difficulty finding a technological consultant appropriately meeting their requirements. As a result, this initiative had to work remotely with a technological team, which created communication challenges (Initiative #14). One initiative hired an out-of-house technological team, which led to delays, misunderstandings, and even errors due to language differences (Initiative #9).

In all, 4 initiatives cited governmental procedures and bureaucracy as a barrier to mHealth utilization (Initiative #2, 14, 15, and 19). Instead of directly approaching the national government, previous initiatives have primarily partnered with the local government in order to bypass the lengthier process required to work with the GVN (Initiative #13). In addition, 4 initiatives noted that it was crucial for project managers to build strong relationships with the GVN and advocate for their mHealth initiatives in order to increase the chances of sustainability (Initiative #4, 5, 11, and 13).

## Discussion

### Challenges to Future Implementation

Vietnam is becoming an increasingly technologically driven and connected society with approximately 1.3 mobile subscriptions per person. Even though many initiatives touted mobile phone penetration as an advantage of mHealth use in Vietnam, mobile penetration is only 65%, meaning a majority of the population owns multiple phone numbers, while 35% of the population does not own a cell phone [53]. While stakeholders believe mHealth may have the potential to reduce disparity in the Vietnamese health care system, there are still populations out of the reach of mHealth. While some initiatives cited high or low acceptability, few initiatives formally evaluated the end user acceptability.

Funding and sustainability are major barriers to future mHealth initiatives. Many of the interviewees reported financial challenges, and only 4 out of 15 completed initiatives were considered *continuing initiatives*. The high percentage of external funding will challenge future initiatives, as after external funds are utilized the technological systems built still need to be continually monitored and upgraded in accordance with the changing needs of the end users. Initiatives in Vietnam are dependent on monetary constraints. Finally, there is a lack of evidence on the sustainability of these initiatives outside of the intervention context, as many of the initiatives incentivize their participants to use the mobile technology.

Technologically, mHealth in Vietnam had similar strengths and weaknesses identified in reviews of mHealth utilization in other developing countries [16]. Technological infrastructure was a barrier for previous initiatives and will likely challenge future initiatives until sufficient infrastructure is constructed. Technological infrastructure is dependent on the initiative location. Many health clinics, particularly at the commune level, still do not have Internet connections or computers, which limits the expansion of successful projects to locations with the capacity to support these initiatives. The Vietnamese system has yet to standardize data information, making it difficult for mHealth initiatives to integrate its data with the existing system. Therefore, previous initiatives have had to build their technological systems from the ground up. Another technological barrier not mentioned by interviewees was patient privacy and protection. While more mHealth initiatives in the world are moving toward SMS encryption for increased patient protection [54,55], Vietnamese initiatives did not mention cell phone and SMS encryption, which likely are still at their early stages of development in Vietnam.

Multiple initiatives indicated governmental procedures and bureaucracy were barriers to mHealth utilization. To date, the GVN has shown little intention to foster a legislative environment conducive to mHealth initiatives. In 2010, the prime minister approved an information technology Master Plan aiming to “transform Vietnam into an advanced ICT country by 2020” [56]. The GVN aims to improve infrastructure, human resources, and access related to information communication technology; the GVN has current plans to build a national eHealth architecture and data standards [42].

Despite these ambitions, there is currently still no information technology framework or electronic medical record standardization in Vietnam, which creates discontinuous care and difficulty for mHealth initiatives to connect to existing health data within the existing infrastructure. While this Master Plan gave a general framework for the advancement of eHealth in Vietnam, there is no current legislation relating to mHealth or data security, with the only related policy document having general statements about technical standards for government agencies and one line defining Web-based health activity [57-59].

Thus far, the GVN has focused their efforts on eHealth, or the use of information and communication technologies for health [60], on telemedicine, or the delivery of health care services using communication technology [61]. In 2013, the Ministry of Health passed a decision creating telemedicine networks aiming to train providers in more rural hospitals. As of 2017, the 3 largest telemedicine systems built in Vietnam are the Viet Duc Hospital with 6 satellites, Bach Mai Hospital with 9 satellites, and 108 Military Central Hospital with 8 satellites [62]. Other than this telemedicine initiative, the GVN has shown little support for mHealth initiatives through implementation of their own mHealth projects or through the funding of other projects.

The lack of support from the GVN can be understood, as there are competing health care priorities and lack of a strong mHealth evidence base. First, the GVN is challenged by an overburdened health care system, creating many competing health care priorities, which is the largest barrier to mHealth implementation worldwide [7]. An interview with an employee from the Department of Information Technology of the Ministry of Health stated the current focus for the near future is to write, regulate, and standardize medical records; GVN currently has a pilot project to standardize electronic clinical documents but no imminent plans to support mHealth. Second, the GVN likely does not see any concrete evidence about the effectiveness of mHealth initiatives in Vietnam. Thus far, there is a lack of successful mHealth initiatives in Vietnam, with even the largest of projects failing to reach a scale-up phase due to lack of funding and support.

A lack in mHealth investment is likely part of the GVN’s informal strategy of risk reduction, as they would prefer more rigorous evidence before investing time and money into the field. While some initiatives have attempted to publish results and engage with the GVN, most initiatives have not prioritized information dissemination, as evidenced by the lack of published mHealth results in Vietnam. To date, mHealth initiatives in Vietnam are sporadic and disjointed. There are no current mHealth networks, workshops, or conferences where mHealth stakeholders may share best practices. To the best of our knowledge, the only educational institution working on training individuals for the future of eHealth and mHealth in Vietnam is the Hanoi University of Public Health, which offers an option for students to receive a bachelor’s degree in public health with a specialization in Health Informatics.

### Future Opportunities

While the government has not actively promoted the use of mHealth initiatives, the GVN is not inherently opposed to mHealth utilization. The government seems to be willing to engage with mHealth initiatives, evidenced by the GVN’s previous participation in advisory committees, result dissemination conferences, and examination of previous initiative results. In order to encourage governmental involvement, initiatives must share their results with each other and the GVN, which may encourage the GVN to fund the scale-up of current initiatives.

As technological infrastructure builds, the Vietnamese health care system improves, and prices for these technologies continue to decrease, the GVN might turn more of its attention to mHealth initiatives. In addition, the GVN has consistently supported telemedicine projects. In the future, to ensure the sustainability of mHealth initiatives in Vietnam, more projects must be catalyzed from within the government and from local organizations, as opposed to foreign donors.

Collaboration was an important aspect of mHealth initiatives. Some initiatives were able to bring together expertise from diverse fields. A byproduct of having diverse actors with expertise in different fields was the potential for capacity improvement, especially for the locations that the initiatives piloted their projects. Even if the initiative was not sustained for a long period of time, the intervention period often introduced new resources to the local community. In future, it will be important to maintain collaboration between diverse actors.

Finally, there has already been evidence of mHealth initiatives in Vietnam adopting alternative financing models. For example, one study examined the feasibility and willingness-to-pay for an mHealth-based antiretroviral adherence support system, finding patients who would be willing to pay for these services [63]. Other previous initiatives have attempted to make businesses out of their mobile technology, hoping to raise capital in order to create a sustainable initiative. While current mHealth initiatives have already demonstrated promising opportunities for alternative models of funding, such as social entrepreneurship or private business models, there have yet to be sustainable mHealth initiatives outside of those funded by external donors. As technology improves and evidence increases, there will be many more opportunities for smaller organizations to thrive in the rapidly growing Vietnamese health care system.

### Limitations

Our study has several limitations. Information analyzed in this study was limited to the information available to the authors. There were initiatives that we were unable to obtain information about, and there were likely smaller initiatives that were not discovered by our search methodology. As we were in the process of writing and revising the paper, new initiatives started, and more results were likely published after our primary data collection period. The openness and accessibility of key informants differed, and the qualitative results were based on opinions of key informants. Finally, the methodology used cannot provide results about mHealth’s acceptability or ability to provision higher quality care in Vietnam.

### Conclusions

These results provide important insights that are unique to Vietnam but have broad implications for mHealth worldwide. Our findings suggest the largest advantage of mHealth in Vietnam is its ability to reach hard-to-reach populations and vulnerable groups. On the other hand, mHealth implementation in Vietnam has been challenged by factors including diacritic marks in the Vietnamese language, a significant portion (35%) of the total population lacking cell phone ownership, sociocultural factors relating to privacy, and lack of technological infrastructure. Looking toward the future, the biggest threats to mHealth in Vietnam are related to the absence of government policy, uncertainty of government support, and heavy dependence on foreign funding. In conclusion, while current mHealth initiatives have already demonstrated promising opportunities for alternative models of funding, such as social entrepreneurship or private business models, sustainable mHealth initiatives outside of those funded by external donors are yet to be undertaken. mHealth is an emerging field in Vietnam and should be more rigorously studied in order to examine its effectiveness and guide future mHealth initiatives.

## Appendix

Multimedia Appendix 1 Initiative summaries.

## Abbreviations

ASD: autism spectrum disorder
ECG: electrocardiogram
GVN: Government of Vietnam
SMS: short message service
TB: tuberculosis
